# Neurodevelopmental disorders, like cancer, are connected to impaired chromatin remodelers, PI3K/mTOR, and PAK1-regulated MAPK

**DOI:** 10.1007/s12551-023-01054-9

**Published:** 2023-04-01

**Authors:** Ruth Nussinov, Bengi Ruken Yavuz, M Kaan Arici, Habibe Cansu Demirel, Mingzhen Zhang, Yonglan Liu, Chung-Jung Tsai, Hyunbum Jang, Nurcan Tuncbag

**Affiliations:** 1 Computational Structural Biology Section, Frederick National Laboratory for Cancer Research, Frederick, MD 21702 USA; 2 Department of Human Molecular Genetics and Biochemistry, Sackler School of Medicine, Tel Aviv University, 69978 Tel Aviv, Israel; 3 Graduate School of Informatics, Middle East Technical University, Ankara, Turkey; 4 Department of Chemical and Biological Engineering, College of Engineering, Koc University, 34450 Istanbul, Turkey; 5 Cancer Innovation Laboratory, National Cancer Institute, Frederick, MD 21702 USA; 6 School of Medicine, Koc University, 34450 Istanbul, Turkey

**Keywords:** Chromatin, Cell cycle, Autism, RASopathies, ASD

## Abstract

Neurodevelopmental disorders (NDDs) and cancer share proteins, pathways, and mutations. Their clinical symptoms are different. However, individuals with NDDs have higher probabilities of eventually developing cancer. Here, we review the literature and ask how the shared features can lead to different medical conditions and why having an NDD first can increase the chances of malignancy. To explore these vital questions, we focus on dysregulated PI3K/mTOR, a major brain cell growth pathway in differentiation, and MAPK, a critical pathway in proliferation, a hallmark of cancer. Differentiation is governed by chromatin organization, making aberrant chromatin remodelers highly likely agents in NDDs. Dysregulated chromatin organization and accessibility influence the lineage of specific cell brain types at specific embryonic development stages. PAK1, with pivotal roles in brain development and in cancer, also regulates MAPK. We review, clarify, and connect dysregulated pathways with dysregulated proliferation and differentiation in cancer and NDDs and highlight PAK1 role in brain development and MAPK regulation. Exactly how PAK1 activation controls brain development, and why specific chromatin remodeler components, e.g., BAF170 encoded by *SMARCC2* in autism, await clarification.

## Introduction

Clinically, neurodevelopmental disorders (NDDs) and cancer are vastly different. From the standpoint of cell life, they are tightly linked, albeit in adversarial ways. The cell cycle is motorized by two major incoming signals. One controls cell division primarily via MAPK/ERK (mitogen-activated protein kinase/extracellular signal-regulated kinase) and the Hippo (via YAP/TAZ) pathways. The cell growth is mediated by PI3K/AKT (phosphoinositide 3-kinase/protein kinase B) and Wnt/β-catenin pathways. All are related to cancer (Nussinov et al., 2016a, 2017; Nussinov et al., 2016b) and to NDDs (Caracci et al., 2021), raising the enigmatic question of exactly how the same pathways, proteins, and mutations can lead to these distinct clinical manifestations. NDDs result from dysfunction of the nervous system during embryo development. They may have emerged from dysregulation of neuron differentiation, or evolve during other critical neurodevelopmental stages, such as synapse formation and maturation (America’s Children and the Environment, 2022; Nussinov et al., 2022d; Parenti et al., 2020; Sahin and Sur, 2015; Song et al., 2019; Zhang et al., 2020). The development of the central nervous system in the embryo where malformations may occur encompasses a series of critical processes (Park and Saint-Jeannet, 2010). These include the production of neurons from progenitor cells, the determination of the phenotypes of the neurons, the migration of the newly formed neurons into their positions in the brain, and the formation of the specific synaptic contacts, resulting in precisely wired neuronal circuits. Defects in this complex process can result in NDDs.

**Fig. 1:**
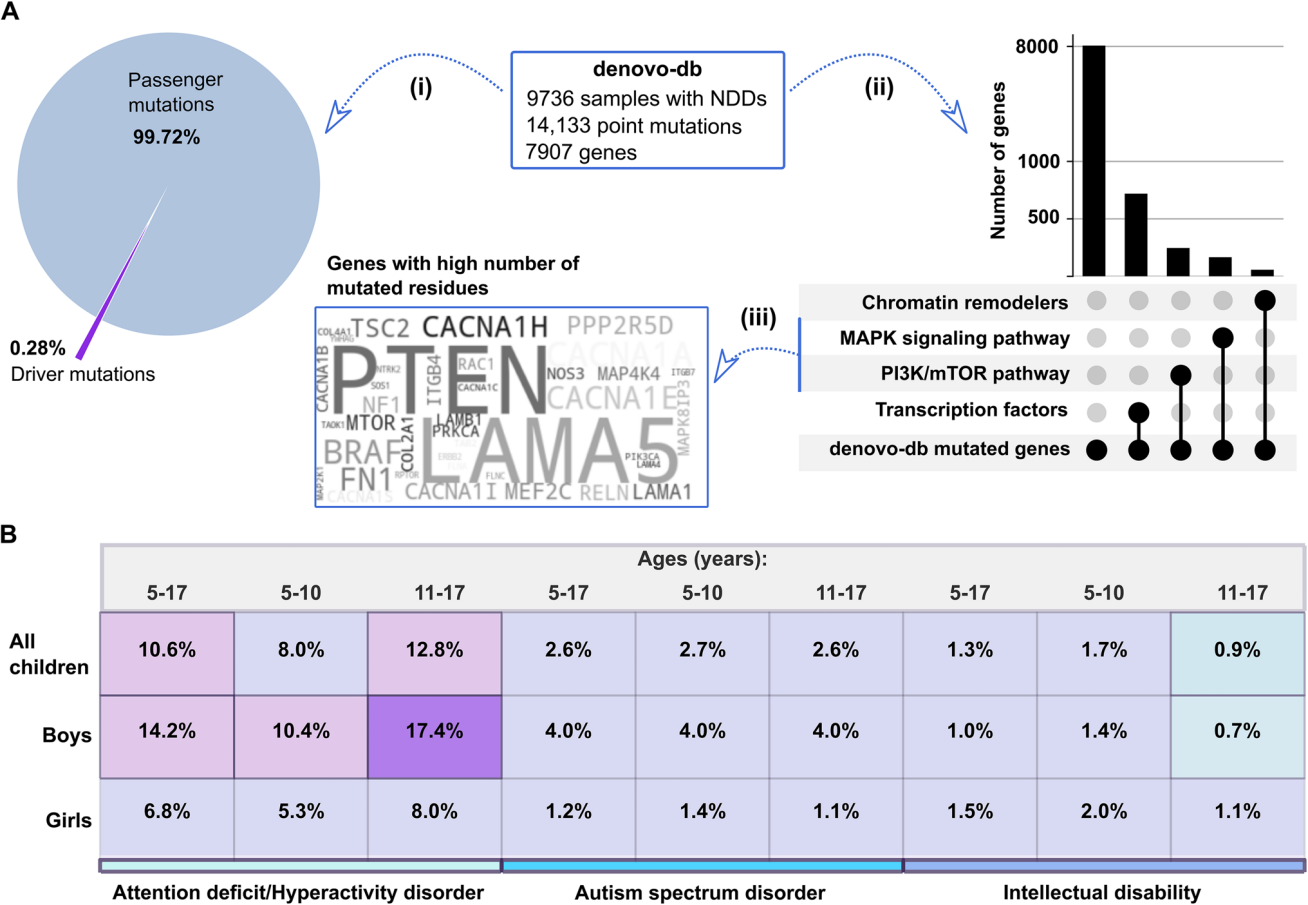
General statistics from genomic and epidemiological studies. **A** denovo-db (https://denovo-db.gs.washington.edu/denovo-db/) deposits mutation profiles of 9736 samples from 20 different neurodevelopmental disorders (NDDs), including attention-deficit/hyperactivity disorder (ADHD), autism spectrum disorders (ASD), intellectual disability, cerebral palsy, etc. These samples have 14,133 point mutations (missense and nonsense mutations) on 7907 genes, where only (i) 0.28% of them are driver mutations (Cancer Genome Interpreter provides 5307 oncogenic mutations). (ii) The upset plot shows the number of genes from different categories among all mutated genes in denovo-db. A black dot represents that the bar includes information about the category described on the left; if two black dots are connected, the corresponding bar size gives the number of genes belonging to both categories on the left. Among 7907 mutated genes, the number of transcription factors and chromatin remodelers is 712 and 50, respectively. Similarly, the proportion of the genes belonging to PI3K/mTOR and MAPK pathways are 239 and 159, respectively. (iii) Gene cloud plot shows some of the genes from PI3K/mTOR and MAPK pathways that have at least three mutated positions on the corresponding protein sequence. The larger font indicates a larger number of mutated residues; for example, *PTEN*, *LAMA5*, and *BRAF* have 12, 11, and 9 mutated residues, respectively. **B** The matrix shows the fraction (%) of the children by age and sex with ADHD, ASD, and intellectual disability between 2016 and 2019, retrieved from America’s Children and the Environment (ACE). ADHD and ASD incidence among the boys are higher than the girls in all age intervals 5–17, 5–10, and 11–17

Here, we connect dysregulated signaling in the MAPK and PI3K/PDK1/AKT/mTOR (where PDK1, phosphoinositide-dependent protein kinase 1; mTOR, mammalian target of rapamycin) pathways with dysregulated cell cycle subverting normal cell proliferation and differentiation, resulting in NDDs or cancer. MAPK is the major pathway in cell division, PI3K/PDK1/AKT/mTOR in cell growth (Torii et al., 2006). Both act in the G1 stage and are required for physiological progression of the cell cycle. Mutations affecting either pathway will damage the normal cell cycle coordination and passage through its stages (Fruman et al., 2017; Vitucci et al., 2013). Mutation strength, the brain cell type, and timing of the expression of the respective gene determine the NDD or cancer outcome (Nussinov et al., 2022b, c). We suggest that the mutations involved in NDDs are weaker than those in cancer, although this may not always be the case, likely since signal strength is determined by additional elements, which can be controlled by the status of chromatin remodeling and gene accessibility (Nussinov et al., 2022c, d). Data suggests that NDDs are relatively common (Frances et al., 2022; Hansen et al., 2018; Udin et al., 1989), likely even more frequent than the statistics indicate (Zhou et al., 2022) (Fig. 1). Some NDD mutations are germline; others emerge during the embryo development along with unidentified background mutational load (Sahin and Sur, 2015). Coupling with inherited germline mutations can result in phenotypic clinical presentation, not observed in the parent (Liljenwall et al., 2022).

Below, we discuss cell proliferation and differentiation, and how mutations can lead to signaling aberrations influencing the critical coordination in the cell cycle resulting in NDDs (Jang et al., 2023; Nussinov et al., 2022e). We connect NDD manifestations and cancer with their respective mutations and the perturbations in cell expression, through the strength of the signaling that the mutations initiate. Chromatin remodeling is an important factor in both cancer and NDDs. NDDs are connected to cell differentiation in embryonic development through cell lineage restriction by chromatin organization and gene accessibility which determine gene expression (Ding, 2015; Maussion et al., 2015). However, not all genes which are involved are protein-coding (Lozano-Urena and Ferron, 2019). This leads us to believe that mutations in chromatin remodelers, which determine gene accessibility, could be more consequential and common in NDDs than in cancer.

### NDDs comorbidity and cancer

NDDs share commonalities (Chow et al., 2019; David et al., 2022; Dewey, 2018; King, 2016; Wagner et al., 2015). They also share commonalities with cancer (Li et al., 2020; Mogavero et al., 2020; Morgan et al., 2021; Nussinov et al., 2022c, d; Qi et al., 2016; Roston et al., 2021; Stephenson et al., 2022; Yang et al., 2021; Yehia et al., 2022). Examples of NDDs include attention-deficit/hyperactivity disorder (ADHD), autism spectrum disorder (ASD), learning disabilities, intellectual disability, cerebral palsy, and damaged vision and hearing affecting speech, motor skills, behavior, memory, and learning. An estimated 15% of children in the USA ages 3 to 17 years are affected. Phenotypic presentations of NDDs typically have more than one of these conditions, for example, ADHD and a learning disability (America’s Children and the Environment, 2022; Pastor and Reuben, 2008) and autism and ADHD (Grupp-Phelan et al., 2007; Kelleher et al., 2000; US Department of Education, 2007). The considerable comorbidity, phenotypic overlap, and genetics suggest that intellectual disability, ASD, ADHD, schizophrenia, bipolar disorder, and other NDDs exist in neurodevelopmental continuum (Morris-Rosendahl and Crocq, 2020). CNVs (copy number variants) in ASD (e.g., *CDH8*, 16p11.2 deletion syndrome, *SCN2A*) and intellectual disability and ASD associations support a genotype connection (Rein and Yan, 2020; Rylaarsdam and Guemez-Gamboa, 2019; Siu et al., 2019). Common underlying pathway dysregulation, as in the case of the Ras/MAPK RASopathies, also exhibit overlapping phenotypic features. However, risk factors, including psychosocial and environmental, can also play a role (America’s Children and the Environment, 2022). The statistics of children with NDDs is relatively high (Fig. 1). From 2011 to 2019, between 1.0 and 1.7% were diagnosed with intellectual disability, increasing from earlier years, likely due to diagnosis tests and setting and adherence to criteria. Between 2014 and 2019, the rates of reported autism ranged from 2.3 to 2.9%. A total of 7.1% of children ages 5 to 17 years had been diagnosed with a learning disability. From 1997 to 2019, the proportion of children ages 5 to 17 years reported to have been diagnosed with ADHD increased from 6.3% in 1997 to 9.9% in 2019.

**Fig. 2:**
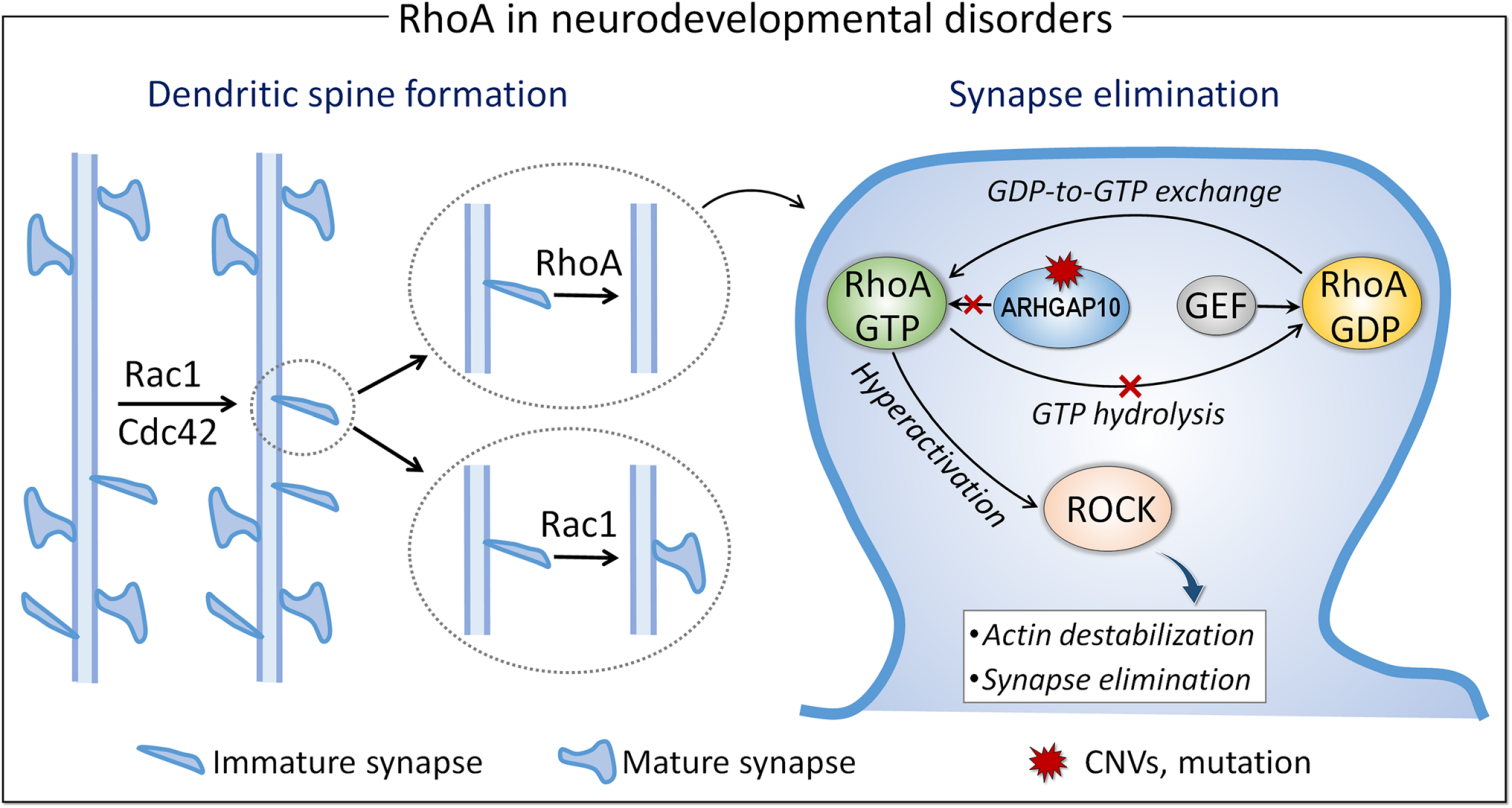
The role of RhoA in neurodevelopmental disorders (NDDs). Hyperactivation of RhoA can induce NDDs as well as cancers. In neural cells, the activation of Rac1 and Cdc42 increases immature spines (Zhang et al., 2021a). Some synapses are eliminated by RhoA-dependent signal, while other synapses will grow into mature forms through Rac1-dependent pathway. RhoA is activated by guanine-nucleotide exchange factors (GEFs) through GDP-to-GTP exchange and deactivated by Rho GTPase activating protein 10 (ARHGAP10) via GTP hydrolysis. A severe psychiatric disorder, schizophrenia, is associated with rare exonic copy number variants (CNVs) in *ARHGAP10* or with both CNVs and a missense variant S490P in the RhoGAP domain of ARHGAP10 (Sekiguchi et al., 2020). In synapse, dysfunction of ARHGAP10 fails to suppress the RhoA activation, resulting in the hyperactivation of RhoA. Hyperactive RhoA activates Rho-associated protein kinase (ROCK) that regulates actin cytoskeleton destabilization, leading to the elimination of synapse

With the immune and nervous systems coevolving as the embryo develops, immunity serves as the link between NDDs and cancer (Nussinov et al., 2022d). NDDs and cancer share proteins, and patients with NDDs have a higher risk of cancer. More than a third of the cancer driver genes have been cataloged as risk genes for NDDs (Nussinov et al., 2022c; Su et al., 2021). NDDs and cancer signal through common cellular pathways, including MAPK and PI3K/ PDK1/AKT/mTOR with phosphatase and tensin homolog (PTEN) (Jang et al., 2021), critical in cell division and growth, thus proliferation and cell differentiation (Crawford et al., 1994; Jang et al., 2023; Qi et al., 2016). Current data suggest that mutations can be shared as well. NDDs and cancer were also shown to be invariably connected with dysregulation of the networks of small GTPases, including Ras (Cirstea et al., 2022), RhoA, Rac, Cdc42, and Rap. Examples include dysregulation of Rho GTPase (Fig. 2), e.g., *ARHGAP10*, a gene for schizophrenia risk, encoding Rho GTPase-activating protein 10 (Sekiguchi et al., 2020), which also plays a role in the proliferation, migration, and invasion of lung cancer cells (Teng et al., 2017), and prostate cancer (Gong et al., 2019). ASD (Amar et al., 2021; Busch et al., 2019; Iakoucheva et al., 2019) and cerebral palsy (Jin et al., 2020) have also been associated with dysregulation of Rho GTPase levels and signaling. PROS (*PIK3CA*-related overgrowth spectrum) [e.g., (Martinez-Lopez et al., 2017; Venot et al., 2018; Venot and Canaud, 2017)] appears associated with cell positioning mediated by Rho GTPase (Torroba et al., 2018), whereas PI3K’s contribution to cell proliferation can take place through a major Ras pathway, PI3K/PDK1/AKT/mTOR, a dominant contributor to cell growth. Cognitive impairment was associated with Rab (Ginsberg et al., 2011). Rab has also been identified as a major regulator of the intracellular positioning and of cell growth, survival, and programmed cell death or apoptosis (Gopal Krishnan et al., 2020). RASopathies (Rauen, 2013), including, e.g., neurofibromatosis type 1 (NF1), Noonan family of syndromes (NS), Costello syndrome (CS), NS with multiple lentigines (NSML, formerly known as LEOPARD syndrome), Legius syndrome (LS), capillary malformation-arteriovenous malformation (CM-AVM) syndrome, and cardio-facio-cutaneous (CFC) syndrome, have been associated with proteins in the Ras/MAPK signaling network, including Ras, Raf, mitogen-activated protein kinase kinase (MEK), ERK, and SHP2 (SH2 domain-containing protein tyrosine phosphatase 2), their regulators, effectors, and components of their signaling pathways (Rauen, 2013). Nodes in the Ras signaling network are all associated with RASopathies and with cancer. Cyclin-dependent kinases (CDKs) were also connected with rare developmental disorders (Colas, 2020). Pathways related to cytokines, toll-like receptors (TLRs), and fibroblast growth factor receptor (FGFR) are also common in NDDs and cancer. TLRs, IL-1 (interleukin-1), GIT1 (ARF GTPase-activating protein GIT1), and FGFR, which activate RhoA (Manukyan et al., 2009; Oda and Kitano, 2006), are also common, acting through Src family kinases and NF-κB (nuclear factor kappa B). NDDs are however associated with expression of mutant proteins encoding germline mutations or mutations that emerge during embryonic development, or deletions of key protein players, as can be in the case of autism (Chau et al., 2021; Urresti et al., 2021). In contrast, mutations associated with cancer are largely sporadic, emerging throughout life.

Recently, we asked how same-gene mutations can lead to both cancer and NDDs and why individuals with NDDs have a higher risk of cancer (Nussinov et al., 2022c). We suggested that the first question can be addressed by considering the mutation strength, the timing windows, and the cell type-specific perturbation levels of the expression of the respective protein, and of proteins in the respective signaling pathway, and their regulators. These latter elements, the expression levels and timing windows, point to the vital role of chromatin reorganization.

Our hypothesis that mutation strength, the timing windows, and the cell type-specific perturbation levels of expression of the respective protein are key factors determining clinical outcome is consistent with observations: As to mutation strength, our statistics indicate that strong hot spots mutations tend to be correlated with cell proliferation in cancer, whereas weak/moderate mutations with NDDs (e.g., see mutation statistics in Fig. 1). As to timing windows, cancer emerges from somatic mutations throughout life, whereas NDDs arise from germline mutations expressed during embryonic development. As to cell type-specific perturbation levels of expression of the respective protein, NDDs are connected to certain brain cell types. Especially, brain cells are not homogeneous, and genes of different cell types can be expressed at different times, influenced by temporal chromatin reorganization during brain development. A recent census of types of cells in the brain (Brain Initiative Cell Census Network, 2021) reported that the number of cell types varies depending upon the method used for sorting them. Still, 25 classes of cells were identified, including 16 different neuronal classes and 9 non-neuronal classes, with each composed of multiple subtypes of cells. Within this framework, as an example, consider that cerebral palsy is caused by abnormal development of part of the brain (or by damage to parts of the brain) that control movement, which likely differs from that of intellectual disability, which relates to a different part of the brain. However, intellectual disability can co-occur with cerebral palsy, suggesting some common or adjoining genes partaking in a common chromosomal deletion, or CNVs as in ASD 16p11.2 deletion. The mutations may be harbored on genes, which in the different cell types, may or may not co-express at the same time window during embryonic brain development.

Chromatin structure, thus gene accessibility, is dynamic, varying during embryo development and between embryonic and adult differentiated cells. Since gene accessibility is a key factor in protein expression (Nussinov et al., 2021c; Zhang et al., 2021b), it is a major determinant of cell lineage and cell types. Consider that for a signal to propagate downstream to activate (or repress, in repressors) expression, the presence of an activating mutation is not enough (Nussinov et al., 2022e). The level of the protein should also be high (Nussinov et al., 2022b). Recall that even without a mutation, a high- (or low-) enough protein level, through, e.g., gene duplication or dysregulation of its level, for example through feedback loops (Nussinov et al., 2022e), can already initiate the signal, drive cancer (Nussinov et al., 2021b), and promote NDDs. As to why individuals with NDDs have a higher risk of cancer, we suggested that since the same genes (and sometimes the same mutations) are involved, preexisting embryonic NDDs mutations already predispose the individual to cancer. Somatic mutations can collaborate with the preexisting weak/moderate chromatin remodeling embryonic mutations, resulting in protein activation of a sufficiently large protein population.

Here, we address questions that relate to the clinical presentation. Among these is why the relatively high statistics of children with NDDs. We delve into the origin of the differences between cancer and NDDs and among NDDs. We discuss the dysregulation of the cell cycle, focusing on cell proliferation and differentiation, connecting them with dysregulated signaling of the MAPK and PI3K/PDK1/AKT/mTOR pathways and NDDs. We also discuss the pivotal role of chromatin remodelers, whose modification in the healthy cell or dysfunction in disease can be decisive in aberrant differentiation, and with chromatin regulators playing a key role, as shown in ASD, intellectual disability, and other brain developmental disorders (Brookes, 2016; Chen et al., 2016; Cotney et al., 2015; Davis, 2023; De Rubeis et al., 2014; Gabriele et al., 2018; Hoffmann and Spengler, 2019; Hsieh and Gage, 2005; Iwase et al., 2017; Larizza and Finelli, 2019; Larrigan et al., 2021; Lasalle, 2013; Lim et al., 2022; Markenscoff-Papadimitriou et al., 2021; Marshall and Brand, 2017; Medrano-Fernandez et al., 2019; Sokpor et al., 2017; Suliman et al., 2014; Tabolacci and Neri, 2013; Yauy et al., 2019; Zhao et al., 2018). This can be understood in the framework of its biophysical properties, making the interactions of its compartments liquid-like and highly dynamic (Belaghzal et al., 2021; Hansen et al., 2021; Itoh et al., 2021; Nussinov et al., 2021a), with the intrinsic chromatin condensates displaying liquid-like material properties (Gibson et al., 2021), but also described as having a solid-like behavior at mesoscales (Strickfaden et al., 2020; Zidovska, 2020). It was also observed to be fluid-like in the crowded nucleus, readily responding to magnetic forces applied to a genome locus, by displacement by several micrometers (Keizer et al., 2022; So and Tanner, 2022).

### Cell proliferation and differentiation, chromatin remodeling, and the cell cycle

During development, cells proliferate and differentiate into specialized cell types (Cooper, 2000). Proliferation results from cell division (MAPK) and cell growth (e.g., PI3K/PDK1/AKT/mTOR pathway) (Kaldis, 2016). Normal cell differentiation results from regulated gene expression, which is largely governed by chromatin remodeling and the consequent gene accessibility (Lopez-Jimenez and Gonzalez-Aguilera, 2022). Cell proliferation increases the number of cells. Differentiation, also acting at the G1 stage, sets their function and influences their morphology. Successive differentiation events are constrained by chromatin organization, which largely determines the cell lineage. Physiological cell proliferation is balanced by apoptosis and differentiation, with progenitor cells differentiating into cell types that belong to the same tissue or organ, e.g., hematopoietic stem cells in the bone marrow can differentiate into blood cells, including myeloid and lymphoid progenitor cells, which can only differentiate into distinct cell types within this blanket. Chromatin pre-organization constrains the evolution of the three-dimensional genome structure during the cell differentiation process (Blanco et al., 2021; Dong and Cheung, 2021). Making compactly packed genes available for transcriptional programs faces energy barriers (Ferreiro et al., 2014, 2018; Maeshima et al., 2020) enforcing an irreversible mammalian cell fate decision (Blanco et al., 2021). Cell lineage proceeds through local chromatin modulation, making the chromatin remodelers and the associated transcription factors highly susceptible to mutations. In differentiating B lymphocytes from a quiescent state, chromosome reorganization in the late G1 phase remains stable through clonal expansion. However, conformational changes were observed in the G1 phase as the cells differentiate, pointing to gene expression (Chan et al., 2021). Chan et al. suggested that a shortened G1 phase might be possible with minor genome restructuring. The recent description of the locally fluidic state of the interactions suggests the feasibility of such reorganization upon some cues.

**Fig. 3:**
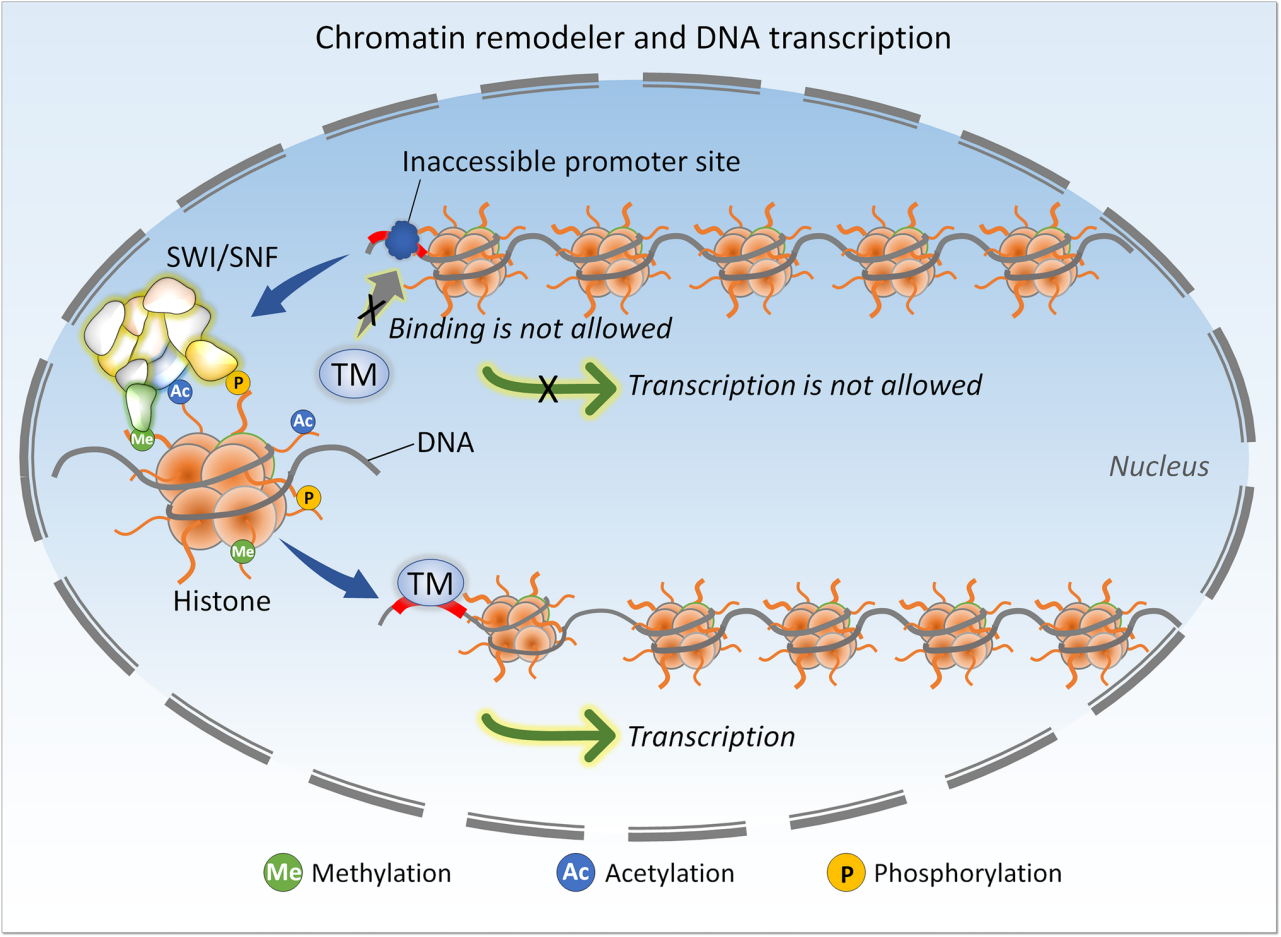
A nucleosome is the basic unit of chromatin, which is formed by DNA wrapping around a histone octamer. This compact structure regulates the stability of genome and prevents its accessibility toward machineries. SWI/SNF (switch/sucrose non-fermentable) is one of chromatin remodeler that regulates DNA transcription. In human cells, SWI/SNF remodeler has three subfamilies, canonical BAF (cBAF), polybromo-associated BAF (PBAF), and non-canonical BAF (ncBAF). To conduct transcription, the wrapped DNA needs to be loosened, and the promotor site should be accessible by machineries. Modifications of histone tails, such as methylation, acetylation, and phosphorylation, mediate tightness of DNA twining around the histone core. Some subunits of SWI/SNF can recognize modifications of histone tails. A catalytic subunit utilizes the energy from ATP hydrolysis to reshape nucleosome, which relaxes the DNA chain and induces the exposure of the promoter site. SWI/SNF remodeler disassembles nucleosome, causing DNA stretching to open the binding site toward transcription machinery (TM). Thereby, the movement of transcription can occur

Remodeling of chromatin structure is a critical factor in gene expression, including cell cycle-associated genes, thus cycle progression (Ma et al., 2015). CDKs drive cell cycle entry. Precursor cells divide prior to becoming fully differentiated. Differentiation, thus lineage, is constrained by chromatin accessibility. Full differentiation is coupled with proliferation arrest and permanent exit from the cell cycle (Ruijtenberg and van den Heuvel, 2016). Chromatin remodelers, such as SWI/SNF (switch/sucrose non-fermentable) complexes (Alver et al., 2017), collaborate with transcription factors to regulate the cell cycle and execute the cell type-specific gene expression (Fig. 3), which coordinate cell cycle exit with terminal differentiation. Among these is the chromatin-remodeling complex Brahma-related gene 1 (BRG1)-associated factor (BAF), a SWI/SNF component (Barutcu et al., 2016; Ronan et al., 2013). BAF is a nonspecific minor groove phosphate backbone DNA-binding protein that can cross-bridge two double-stranded DNA segments, thereby contributing to chromatin compaction (Marcelot et al., 2021). It also binds nuclear envelope proteins bridging the inner nuclear membrane and the nucleoskeleton formed by lamins. Despite minor-groove nonspecific binding, the multi-subunit BAF complex with histone-/DNA-binding domains only binds a subset of genomic DNA sites (Ho et al., 2019). Although recently increasingly elucidated, the details of the complex mechanism explaining this selectivity are still not entirely clear. BAF also binds multiple transcription factors, some of which can recruit it (Marcelot et al., 2021). Mutations in BAF can promote NDDs including intellectual disability, developmental delay (Kosho et al., 2014; Santen et al., 2013; Van Houdt et al., 2012; Vandeweyer et al., 2014), autism (Neale et al., 2012), and schizophrenia (Loe-Mie et al., 2010) [for comprehensive descriptions, see Machol et al. (2019) and references therein]. The changes in accessibility are dependent on the subunit that is lost (Schick et al., 2019). Accessibility is reduced in *ARID1A*, *SMARCC1*, and *SMARCA4* knockout cells and is higher in *ARID1B* mutants. BAF170, a common core BAF subunit, is encoded by *SMARCC2*. *SMARCC2* is among the high-confidence candidates involved in regulating ASD (Ben-David and Shifman, 2013), intellectual disability, and developmental delay. Carriers of these disorders often have missense variants in the SWIRM (SWI3, RSC8, and MOIRA) and SANT (SWI3, ADA2, N-CoR, and TFIIIB) domains of BAF170. Function-wise, the SWIRM domain binds di-nucleosome structures and is involved in protein-protein interactions related to gene expression (Aravind and Iyer, 2002). The SANT domain appears to function as a histone tail in DNA binding (Yoneyama et al., 2007). Disordered tails protrude from the DNA-wrapped core and are critical in chromatin regulation (Ghoneim et al., 2021). Members of the ATP-dependent CHD (chromodomain helicase DNA-binding) family of remodelers have tandem chromodomains in the N-terminal region and a central SNF2-like ATPase domain. Many of them have clear NDD links and etiology (Alendar and Berns, 2021). They are also involved in the dynamic regulation of chromatin accessibility. Mutations in these helicases can promote DNA copy number alterations leading to aberrant expression in NDDs and cancer.

The antagonism of cell proliferation and differentiation influences the clinical outcome. Strong driver mutations in transcription factors lead to uncontrolled cell proliferation in cancer. Weak mutations in chromatin remodelers and transcription factors are associated with dysregulated differentiation in NDDs. The more sluggish expression of cell cycle-associated genes can extend the time the cell spends in the G1 phase, influencing cell differentiation and NDDs clinical presentation. In rapid proliferation, G1 is short. PI3K, Myc, E2F (a transcription factor), and CDK2 are hyperactivated in embryonic stem cells in high serum or in the presence of leukemia inhibitory factor, and MAPK, CDK4, p16 family, p21 family, and retinoblastoma protein (pRb) are inhibited, leading to such an outcome (Li and Kirschner, 2014). Differentiation follows inhibition of the cell cycle. Initiation of differentiation coincides with cell cycle arrest; terminal differentiation is linked to the G1/S transition. In healthy somatic cells, cell cycle-associated factors interact with transcription factors such as MyoD (myoblast determination protein 1), which regulates the expression of muscle-related genes, committing undifferentiated cells to the muscle. Upon cell cycle exit, the transcription factors activation is terminated, with a shift toward insulin signaling, maintaining glucose homeostasis, away from survival and growth. In proliferating cancer cells, the coordination between proliferation and differentiation is damaged (Ballabeni et al., 2011; Hanahan and Weinberg, 2011; Li and Kirschner, 2014; Ruijtenberg and van den Heuvel, 2016). However, overexpression of p21^Cip1^ (cyclin-dependent kinase inhibitor 1), p27^Kip1^ (cyclin-dependent kinase inhibitor 1B), p14^ARF^ (ARF tumor suppressor), and p16^INK4A^ (cyclin-dependent kinase inhibitor 2A) cell cycle inhibitors was observed to not only inhibit proliferation but be sufficient for inducing differentiation in some cancer cells (Adachi et al., 1997; Kranenburg et al., 1995; Matushansky et al., 2000). In the Drosophila wing, changes in chromatin accessibility of cell cycle genes result in cell cycle exit during terminal differentiation (Ma et al., 2019b).

Transcription factors can stimulate differentiation and lead to cell cycle arrest, and this double action has been exploited to treat leukemia (Rosenbauer and Tenen, 2007). Whereas an increase in the expression levels of most transcription factors increases the frequency of transcriptional bursts of the genes they regulate, c-Myc’s overexpression was observed to increase the duration rather than the frequency (Patange et al., 2022). Surprisingly, variations in Myc’s dwell time on the order of seconds result in changes on the order of minutes in transcription duration burst time. How the extended duration impacts transcription is still unclear, although it was suggested that Myc changes the binding dynamics of transcription factors involved in RNA polymerase II.

### PI3K/mTOR and MAPK pathways are connected, complementary, and critical in proliferation and differentiation

**Fig. 4:**
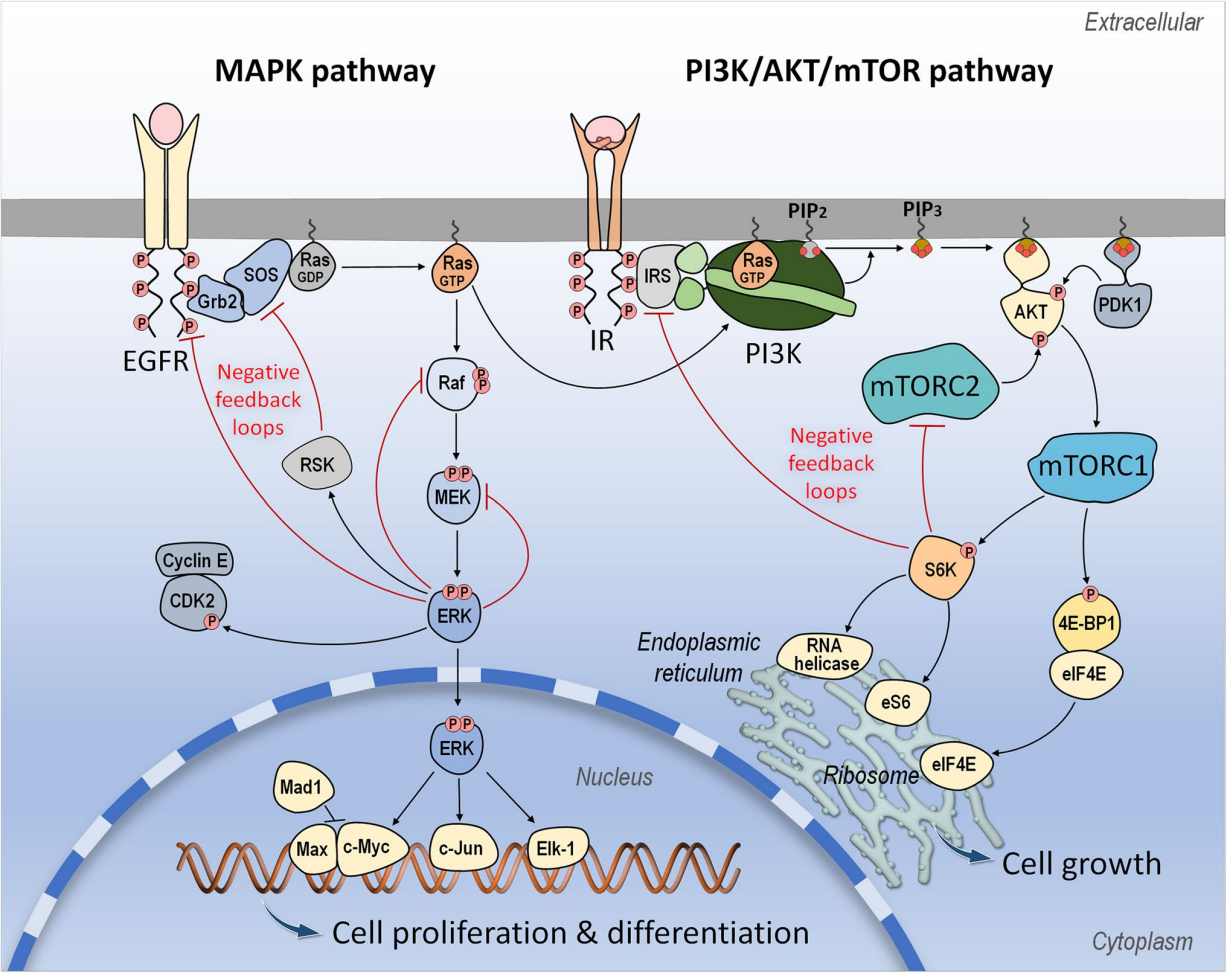
Crosstalk between MAPK and PI3K/AKT/mTOR pathways for cell proliferation, differentiation, and growth. Extracellular stimulation of epidermal growth factor receptor (EGFR) recruits the growth-factor receptor bound protein 2 (Grb2) and guanine nucleotide exchange factor (GEF), i.e., Son of Sevenless (SOS), to activate Ras. The activated GTP-bound Ras promotes the activations of the MAPK and PI3K/AKT/mTOR pathways. In the MAPK pathway, ERK is located at the bottom of the pathway, which translocates into the nucleus and activates the transcriptional factors including c-Myc, c-Jun, and Elk-1, leading to cell proliferation and differentiation. It also phosphorylates CDK2 to regulate cell cycle. The lipid kinase, PI3K, is activated by the insulin receptor (IR) and insulin receptor substrate (IRS), phosphorylating the signaling lipid PIP_2_ to PIP_3_. AKT is recruited to the PIP_3_-enriched microdomain of plasma membrane and activated by PDK1 and mTOC2. AKT activates mTORC1 that phosphorylates the downstream ribosomal S6 kinase (S6K) and eukaryotic translation initiation factor 4E (eIF4E)-binding protein 1 (4E-BP1). The ribosomal protein S6 (eS6) and RNA helicase are activated by phosphorylated S6K, and the eIF4E is released from the phosphorylated 4E-BP1, promoting the translation initiation and elongation for cell growth. Both pathways are regulated by the negative feedback loops (red lines). ERK may inhibit Raf and MEK, decreasing ERK’s activation in the MAPK pathway. ERK activates RSK that phosphorylates SOS1, negatively regulating the MAPK pathway. ERK also phosphorylates EGFR, downregulating EGFR signaling. Phosphorylations of IRS and Rictor in mTORC2 by S6K decrease AKT and mTORC1 signaling in the PI3K/AKT/mTOR pathway

For the cell to proliferate, both Ras/ERK (MAPK) and PI3K/mTOR pathways are essential. Neither pathway is a linear phosphorylation cascade. They are connected, they crosstalk, and they are complementary (Fig. 4). They regulate each other through feedback loops and coregulate cell functions. Both feed into the cell cycle (Mendoza et al., 2011). ERK is at the bottom of the MAPK (Mebratu and Tesfaigzi, 2009). Under physiological conditions, its activation initiates through stimulation of RTKs (receptor tyrosine kinases) or GPCRs (G protein-coupled receptors) which recruit adaptor protein Grb2 (growth factor bound protein 2) and on to GEF (guanine nucleotide exchange factor, e.g., SOS, Son of Sevenless). Ras activation by SOS leads to activation of Raf, which in turn activates MEK1/2. These series of events are at the membrane. Allostery acts to relieve their autoinhibition. MEK1/2 phosphorylates ERK1/2 on both threonine and tyrosine. ERK phosphorylation of MEK negatively regulates MAPK. Phosphorylated ERK1/2 translocate into the nucleus within 15 min of activation. There ERK1/2 along with ribosomal S6 kinase (S6K) phosphorylate transcription factors leading to cell type-specific protein synthesis (Mebratu and Tesfaigzi, 2009). The activity peaks at 5–10 min after activation. This is followed by a 6-h long second wave of lower activity, lasting until the late G1 cell cycle stage (Kahan et al., 1992; Meloche, 1995; Meloche et al., 1992; Yamamoto et al., 2006). Signal strength is a key determinant of the outcome. If the signal is strong and sustained, the likely outcome is cell proliferation. Translocation to the nucleus is required for the G1 to S (synthesis) cell cycle progression (Brunet et al., 1999; Cheng et al., 1998; Jones and Kazlauskas, 2001; Treinies et al., 1999). ERK1/2 are inactivated during the G1/S passage (Meloche, 1995). Nuclear translocation of ERK1/2 dimer is helped by integrin-mediated organization of the actin cytoskeleton (Aplin et al., 2001; Danilkovitch-Miagkova et al., 2000), and the nuclear pore complex (Adachi et al., 1999; Khokhlatchev et al., 1998; Kondoh et al., 2005; Matsubayashi et al., 2001; Whitehurst et al., 2002), although the monomer can diffuse passively. Cytosolic ERK1/2 inhibit survival and proliferation and mediate proapoptotic proteins, promoting cell death. ERK also regulates Thr160 phosphorylation of cyclin-E/CDK2 (Lents et al., 2002). It collaborates with AKT to phosphorylate Myc and increase its expression, critical for cell cycle entry, and repress p27^kip1^ (Chambard et al., 2007). ERK activity promotes the proliferation of muscle myoblasts and the terminal differentiation of myocytes (Michailovici et al., 2014). The subcellular localization of ERK determines whether it stimulates skeletal muscle proliferation or differentiation. ERK1/2 phosphorylation is required for early neuronal differentiation and survival of embryonic stem cells (Li et al., 2006).

The PI3K/PDK1/AKT/mTOR is also a phosphorylation pathway cascade (Fig. 4), and it influences the cell cycle at the G1 phase. It is a cell growth pathway. To divide, cells must first reach a critical size. None of the kinases that compose it cross the membrane into the nucleus. Instead, the mTOR complex 1 (mTORC1) regulates cell growth through phosphorylation with its substrates involved in protein synthesis, including the eukaryotic translation initiation factor 4E (eIF4E)-binding proteins (4E-BPs) and S6K1/2 (Cargnello et al., 2015). 4E-BP phosphorylation inhibits its binding to eIF4E, allowing the initiation of translation. Phosphorylated S6K acts on transcription factors, the ribosomal protein S6, RNA helicases, and additional proteins acting in translation initiation and elongation (Mendoza et al., 2011; Sengupta et al., 2010). Thus, PI3K regulates the cell cycle through AKT, mTOR, and S6K. The mTOR inhibitor rapamycin action on G1 cell cycle progression resembles the inhibition exerted by cyclin-D1, CDK4, and pRb phosphorylation. Hence, PI3K promotes G1 cell cycle progression and cyclin expression through its pathway (Gao et al., 2003). Growth rates are higher in small cells and lower in large cells (Ginzberg et al., 2018). Cell size and mass are controlled by cell cycle progression and the PI3K/mTOR pathway through its S6K1 and 4E-BP1/eIF4E substrates. Cell growth is critical for sustained cellular proliferation (Fingar et al., 2002). Inhibition of mTOR/S6K signaling results in reduced cell size (Fumarola et al., 2005).

The intensity and duration of pathway activation are regulated by the strength of the stimulus and by feedback loops (Mendoza et al., 2011). In disease, dysregulated Ras/ERK signaling can take place through a combination of multiple mutations and overexpression. Strong driver mutations in these pathways, as well as in transcription factors, lead to uncontrolled cell proliferation in cancer; moderate/weak mutations in these pathways, chromatin remodelers, and transcription factors are associated with dysregulated differentiation in NDDs. A combination of very strong hotspot driver mutations or overexpression can lead to oncogene-induced senescence (OIS) (Lemmon and Schlessinger, 2010; Meloche and Pouyssegur, 2007). At the same time, mutation strength does not necessarily imply highly potent signaling (Nussinov et al., 2022e). Negative feedback loops can depress Ras/ERK and PI3K/mTORC1 signaling (Fig. 4). Through phosphorylation, ERK can inhibit Raf and MEK1, decreasing ERK’s activation, providing one example for the Ras/ERK pathway (Dhillon et al., 2007). S6K phosphorylation of insulin receptor substrate protein and Rictor decreases AKT and mTORC1 signaling, providing an example for PI3K/mTORC1 pathway (Dibble et al., 2009; Julien et al., 2010; Sengupta et al., 2010; Treins et al., 2010). A third example concerns c-Myc. c-Myc is a transcription factor that can bind to Max to promote growth and survival. The Mad1 transcription factor competes with Max for c-Myc, which depresses transcription. c-Myc is a highly unstable protein, functioning as an obligate heterodimer with Max to bind DNA and perform its oncogenic activity. Phosphorylation of newly synthesized c-Myc protein at position Ser62 is mediated by ERK, resulting in c-Myc stabilization (Sears et al., 2000).

**Fig. 5:**
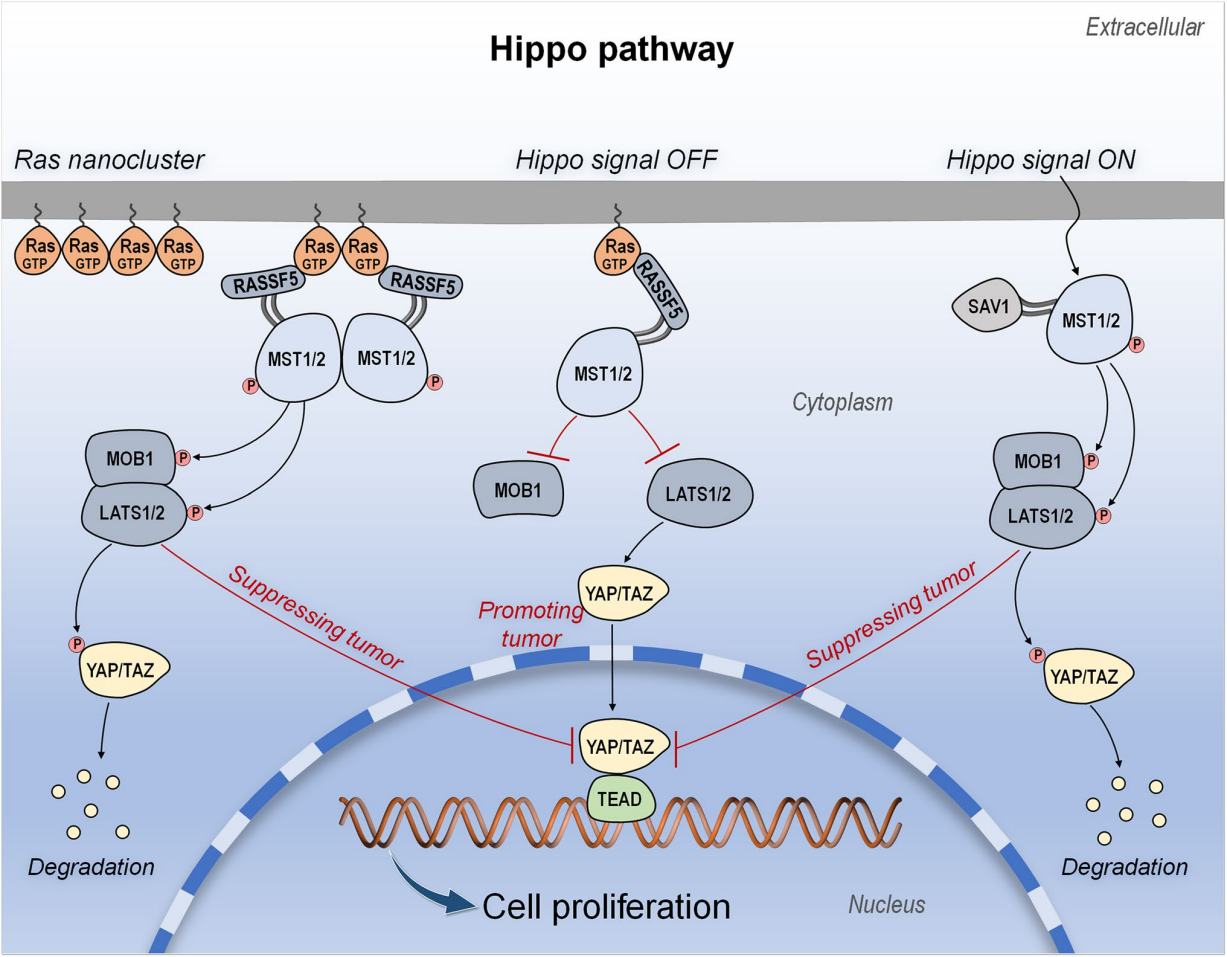
The Hippo pathway. Acting as a tumor suppressor, Ras association domain family 5 (RASSF5) coupled with Ras dimer and the Hippo pathway inhibit cell proliferation. RASSF5 is an adaptor protein, promoting dimerization of mammalian sterile 20-like kinase 1/2 (MST1/2). Cross-phosphorylated MST1/2 by each kinase domain phosphorylates MOB kinase activator 1 (MOB1) and large tumor suppressor 1/2 (LATS1/2), leading to phosphorylation of YAP/TAZ (Yes-associated protein/transcriptional coactivator with PDZ-binding motif) proteins. Phosphorylation of YAP/TAZ stimulates proteolytic degradation. In the absence of Hippo signal, the unphosphorylated YAP/TAZ translocate into the nucleus to stimulate the transcription factor TEAD to activate downstream target genes, leading to cell proliferation. In the presence of Hippo signal, MST1/2 in complex with a scaffolding protein, salvador homolog 1 (SAV1), is phosphorylated by multiple upstream signals, leading to phosphorylation cascade to YAP/TAZ and resulting in proteolytic degradation

Even though here we focus on MAPK and PI3K/mTOR signaling cascades and the impact of their dysregulation, we note that the Hippo signaling pathway (Fig. 5) similarly feeds into the cell cycle and has been implicated in cell development and cancer (Fu et al., 2022; Ma et al., 2019a; Zheng and Pan, 2019). Like MAPK, the Hippo pathway, especially YAP/TAZ (Cunningham and Hansen, 2022), is critical in cell division, thus may rescue debilitating MAPK inhibition, collaborating with PI3K/mTOR in cell proliferation (Nussinov et al., 2016b).

Thus, the critical factor is the number of active molecules, not the mutation strength (Nussinov et al., 2022b). A stronger mutation will lead to more molecules being activated as compared to a moderate/weaker mutation. However, a high expression level can increase the population of active molecules harboring moderate mutations, strengthening the signal, and negative feedback loops may lower the population of active molecules harboring strong mutations, dampening the emitted signal. Thus, a better measurement of the transformation potential is the number of activated molecules rather than the mutation strength.

### PAK1 is a key autism candidate gene that also regulates the MAPK pathway

The p21-activated kinase 1 (PAK1), an effector of both Rac1 and Cdc42 RhoGTPases (Leone et al., 2010), is a critical kinase in the cell. Cdc42 has a key role in the polarity and proliferation of radial glial cells in the ventricular zone. Rac1 contributes to the normal proliferation and differentiation of progenitor cells in the subventricular zone and in the survival of both progenitors. Progenitor cells in the ventricular zone and in the developing forebrain give rise to neurons and glial cells, clarifying why PAK1 dysregulation can be involved in NDDs. PAKs are critical in cytoskeletal organization in neuronal development as well as synaptic function. Their pro-survival signals control neuronal cell fate (Civiero and Greggio, 2018). PAK1 is active as a monomer. Normally, in neurons, PAK1 dimers are in a trans-inhibited conformation, with the autoinhibitory domain of one monomer covering the kinase domain of the other. Binding to Cdc42 or Rac1 promotes dissociation of the dimers and conformational change. Recent experimental and clinical data and modeling suggested that PAK1 variants can interfere with the trans-inhibition of PAK1 dimers, reducing autoinhibition, enhancing the active monomeric state, autophosphorylation, and activation. This was proposed to influence neurite outgrowth, leading to moderate-to-severe intellectual disability, macrocephaly caused by the presence of macrocephaly and ventriculomegaly, with the larger ventricles. This can occur when cerebrospinal fluid is trapped in the spaces, causing them to grow larger, promoting seizures and autism-like behavior (Horn et al., 2019). PAK1 regulates inhibitory synaptic strength. It is a potent, positive regulator of GABA (gamma-aminobutyric acid) transmission, independent of actin regulation. PAK1 inhibitors can rescue some deficiencies associated with NDDs, including the neurofibromatosis model of autism, fragile X syndrome, and schizophrenia (Xia et al., 2018). In cancer, PAK’s overexpression contributes to proliferation. It was suggested that this involves OIS, cell cycle arrest at the G1/S phase, and downregulation of cyclin-A, cyclin-D1, and cyclin-E (Du et al., 2016), especially in cancers arising from PAK1-expressing tissues, such as brain, pancreas, colon, or ovary (Grebenova et al., 2019).

Cellular pathways crosstalk (Liu et al., 2021), and PAK1 may mediate it between MAPK and PI3K/AKT. PAK1 is regulated by PI3K (Chan et al., 2008). PI3K regulates the activation of RhoGEFs that can activate Rac, AKT, and PAK1 (Fruman et al., 2017). PAK1 can activate MAPK (phosphorylates Raf1 at Ser338, MEK1) (El-Baba et al., 2014; Jin et al., 2005; Magliozzi and Moseley, 2021; Qing et al., 2012; Tse and Ching, 2014; Yao et al., 2020). PAK1 phosphorylates MEK1 at Ser218/Ser222 (Wang et al., 2013) and Ser298 (Slack-Davis et al., 2003), and MEK1 activates ERK promoting fibronectin-stimulated MAPK activation (Slack-Davis et al., 2003). In turn, Raf1 can activate PAK1 (El-Baba et al., 2014).

### NDDs pathological manifestations, synaptic impairments, and mutations

NDDs are connected to defects in the patterns of neuronal assembly during development (Batool et al., 2019). Defective patterns are associated with dysfunctional learning, memory, cognition, social behavior, and more (Zoghbi and Bear, 2012). To function, the ensemble of the proliferated and migrated neurons in the developed brain extends their axonal (which are long, unbranched, and presynaptic) and dendritic (short, highly branched, postsynaptic) protein spines to span the gap separating them from their targets. The dendrites receive information. The stimulus signal propagates through dynamic, possibly allosteric, conformational changes to the axon to the target. The alteration of the neurons’ structures upon interaction with the targets is the synapse (Dunn et al., 2018; Liu and Wang, 2014; Matsunaga and Aruga, 2021; Pelkey et al., 2007). The chemical signal is transferred between neurons through interactions. The synapse connections are “plastic,” changing with the environment. While these processes take place throughout life, they are especially impactful during brain development. Regulation of neuronal structural changes is critical for proper neuronal migration, maturation, and synapse formation. Mechanistic details are still unclear.

Mutations are suspected to be a common cause of synaptic impairments in neurodevelopmental diseases. Cited examples include epilepsy, intellectual disability, developmental delay, attention deficit-hyperactivity disorder, schizophrenia, bipolar disorder and obsessive-compulsive disorder, tuberous sclerosis, NF1, Angelman syndrome (*UBE3A*), Rett syndrome (*MECP2*), PTEN hamartoma tumor syndrome, and Phelan-McDermid syndrome (*SHANK3*), with more suspected but to date unidentified (Zoghbi and Bear, 2012) [for reviews, see Betancur (2011) and Guang et al. (2018)]. The mutations are in proteins that are critical regulators of synaptic function. In non-syndromic ASD, mutations appear to be rare, possibly due to a lack of identification. In agreement with this, they have been identified in tuberous sclerosis complex and the Angelman syndrome, both with presentations shared with ASD. That ASD and intellectual disability are associated with defective synapse patterns is further supported by the frequent occurrence of mutations in proteins associated with synaptic structure and function (Zoghbi and Bear, 2012). Furthermore, altering the expression of Rho GTPases affects spine formation in developing neurons (Zhang et al., 2021a) (Fig. 2). Rho GTPases are critical in synaptic regulation (Duman et al., 2021). In neurons, the Rho GTPase Rac1 promotes the growth of axons and dendrites and of spines/synapses, whereas RhoA elicits axonal and dendritic retraction and spine/synapse loss (Luo, 2000; Mulherkar et al., 2017). RhoA is a substrate of Cul3 ubiquitin ligase. In autism, inhibition of RhoA rescued dendrite length and network activity phenotypes (Amar et al., 2021). Finally, dysregulation of Rho GTPases plays critical roles in neurodegenerative disorders, including Alzheimer’s disease (Duman et al., 2021). Several transcription factors have been shown to regulate RhoA expression, including c-Myc, Max, and SMAD4 (mothers against decapentaplegic homolog 4) (Schmidt et al., 2022).

## Conclusions

NDDs and cancer are connected (Nussinov et al., 2022d). They share proteins, pathways, and mutations. Their phenotypic presentations are vastly different, although can be still connected. Individuals with NDDs have somewhat higher probabilities of eventually coming down with cancer. Understanding the similar and distinct hallmarks of the two conditions is vastly important and has been attracting increasing attention in the community. Insight into these may help in pharmacological intervention, with the key question being whether drugs used in cancer can also be useful in NDDs (Nussinov et al., 2022f). Here, our aim is to delve deeper into these questions, integrating experimental and clinical data with conceptual grasp and knowledge (Nussinov et al., 2022a).

Cell differentiation and proliferation are key processes in higher organisms. They fulfill complementary functions. Proliferation increases the number of cells. Differentiation is responsible for the functional specializations of the proliferated cells. In a normal cell cycle, the two processes are coordinated. To date exactly how has been unclear. Especially confounding are the relations between these and the distinct clinical presentations. Cell proliferation is a hallmark of cancer. Cell differentiation, largely constrained and driven by chromatin remodeling, is a hallmark of NDDs. It sets the cell lineage, that is, which specialized cells develop from which progenitor cells. Chromatin remodeling is a key factor determining gene expression, which varies across cell types and developmental time windows. Recently, it has been shown that chromatin is fluid-like (Itoh et al., 2021) within the crowded nucleus when probed in a living cell (Keizer et al., 2022). In contrast to the prevailing entrenched view of a dense, entangled chromatin description, recent measurements of the response of chromatin to force applied at a certain genomic location showed that interphase chromatin, which is the phase of the cell cycle in which a typical cell spends most of its life, is liquid-like, with moderate barriers to overcome topological effects. This updated description is consistent with chromatin remodelers and transcription factors, facilely altering the local chromatin during cell lineage and gene expression.

The differential regulation of gene expression leads to changes in the morphology and function of the proliferated cells, with the critical factor in cell differentiation being the expression levels of the cell-specific proteins at the specific differentiation state. Dysregulation of expression of specific proteins, including RhoA/Rac, may underlie synapses malfunction in NDDs. Uncoordinated proliferation/differentiation may underlie microcephaly pathogenesis. Microcephaly may arise from changes in the relative rates of symmetric and asymmetric divisions or in the differentiation of the neuronal cells, both the outcome of cell cycle defects in timing and progression (Siskos et al., 2021). The increased growth rate in stem cells could be part of the reason for the macrocephaly or abnormally large head size (Thomas, 2020). Defective G1/S phase transition during early stages of brain development appears to correlate with brain maldevelopment in ASD (Chen et al., 2015). Despite these apparent associations, exactly how these processes on the molecular, cellular, and organismal levels are connected and regulated is still enigmatic (Engler et al., 2021; Fidan et al., 2021; Gremer et al., 2011; Pantaleoni et al., 2017), and await resolution.

## Data Availability

This article does not contain raw data to share.
